# Circadian rhythms revealed: unraveling the genetic, physiological, and behavioral tapestry of the human biological clock and rhythms

**DOI:** 10.3389/frsle.2025.1544945

**Published:** 2025-06-04

**Authors:** Renée Morin, Geneviève Forest, Pascal Imbeault

**Affiliations:** ^1^Behavioural and Metabolic Research Unit, School of Human Kinetics, Faculty of Health Sciences, University of Ottawa, Ottawa, ON, Canada; ^2^Laboratoire du Sommeil, Département de psychoéducation et de psychologie, Université du Québec en Outaouais, Gatineau, QC, Canada; ^3^Institut du Savoir Montfort, Montfort Hospital, Ottawa, ON, Canada

**Keywords:** genetics, physiology, behavior, human biological rhythms, circadian clock, chronobiology, chrono-intervention, chrononutrition

## Abstract

This narrative review explores the intricate relationship between biological rhythms and the natural cycles of light and darkness, known as circadian rhythms. This review begins with an examination of empirical evidence dating back to 1729, which indicates that a particular plant displayed rhythmic behavior even in complete darkness. It then considers the evolution and significance of internal biological clocks in humans. The pivotal role of circadian rhythms in regulating physiological processes (e.g., sleep-wake cycles, body temperature, and hormone levels) emphasizing their influence on overall health and wellbeing is discussed. This review also highlights the critical importance of maintaining circadian timing alignment with the environment, as desynchronization can lead to a range of adverse outcomes, including cardiovascular, metabolic, and psychological disorders. By integrating the genetic, physiological, and behavioral mechanisms underlying and associated with biological clocks and rhythms in humans, this review aims to provide clinicians and researchers with a comprehensive perspective on the holistic nature of biological rhythms and their implications for health. Furthermore, the concepts associated with Chrono-intervention, such as chrononutrition and chronomedicine, are introduced as promising approaches to optimizing health outcomes by aligning interventions with the body's natural rhythms are introduced. Through this consideration, this review seeks to contribute to a deeper understanding of chronobiology and its potential applications in improving human health and performance.

## Introduction

Due to the earth's rotation around its axis every roughly 24 h, most life forms on this planet experience regular changes in light and temperature. Various species, spanning from cyanobacteria to humans, have developed internal biological clocks to predict and adapt to these daily fluctuations. Consequently, our internal physiological processes and overall function are intricately linked with this natural cycle (Kuhlman et al., 2018). In fact, in 1729, there was the first publication to report evidence for this evolutionary relationship between internal physiology and geophysical cycle. It was discovered that the plant *Mimosa Pudica* had the particularity of opening its leaves during the daytime and closing them at night. This plant maintained said specific behavior even once placed in complete darkness, hence, deprived of all temporal indices usually obtained from the light-dark cycle (Pittendrigh, 1981). The maintenance of this alternating biological rhythmic activity in such experimental conditions suggested that the organism possessed an internal mechanism of rhythmicity. We now have a clearer understanding that biological rhythms are natural periodical cycles of physiology and behavior generated by endogenous biological clocks (i.e., sleep-wake cycles, body temperature, and hormone levels) (Mukherjee and McClung, 2013; Carskadon and Dement, 2011). Most research on biological rhythms initially focused on cycles with a 24-h period, known as circadian rhythms. These rhythms typically follow the light-dark cycle, maintaining a near- 24-h cycle even without external cues. Circadian rhythms are the most extensively studied in relation to human and animal behavior (Koukkari and Sothern, 2007). However, rhythms with shorter or longer periods, such as 90-min or seasonal cycles, are also significant. Rhythms with periods shorter than 90 min are called ultradian rhythms, while those longer than 28 h are referred to as infradian rhythms (Koukkari and Sothern, 2007; Yates and Yates, 2008). Ultradian rhythms are essential for organismal physiology, regulating key biological functions and adaptive processes (Castellana et al., 2018). Together, these three rhythmic domains form a network of oscillations, much like how different chemical pathways operate simultaneously within the same cell or organelle. Although these rhythms share cyclical oscillations, their genetic regulation and physiological responses differ significantly (Castellana et al., 2018). Consequently, this review focuses solely on circadian rhythms, considering these differences, their relevance to human sleep-wake cycles, and the extensive research conducted on the core topics of this article. Circadian (circa, about; diem, day) biological rhythms play a crucial role in aligning physiological processes with a near-24-h cycle, responding to environmental stimuli (i.e., zeitgebers) to synchronize with the external environment. This synchronization is essential, as variations in circadian timing and disruptions in alignment are linked to increased risks of psychological, metabolic, and cardiovascular diseases, as well as heightened workplace accidents due to reduced sleep quality, vigilance, and attention (Arendt, 2010; Lewis et al., 2018). By understanding the non-negligible interplay of genetic, physiological, and behavioral mechanisms underlying chronobiology, we unleash the significance and potential of using such knowledge to increase performance (mental and physical) and learning while also making links with chronobiology and disease. By understanding biological rhythms and making links between their mechanisms and Chrono-intervention (e.g., chrononutrition, chronomedicine), we are transforming the way we treat medical conditions and rethink medical health. Accordingly, the objectives of this review are to (1) underscore the critical relevance of maintaining circadian timing and synchronization to the environment, (2) provide clinicians and other interested individuals with a nuanced perspective of the holistic nature and dynamics of biological rhythms and clocks, fostering awareness of their far-reaching implications for psychological, metabolic, and cardiovascular wellbeing, and (3) identify current knowledge gaps and future research direction (i.e., Chrono-intervention). This review also introduces fresh figures, enhancing relevance and currency, while incorporating practical clinical insights.

## Chronobiology: nature of circadian rhythms and basic principles

**Figure 1:**
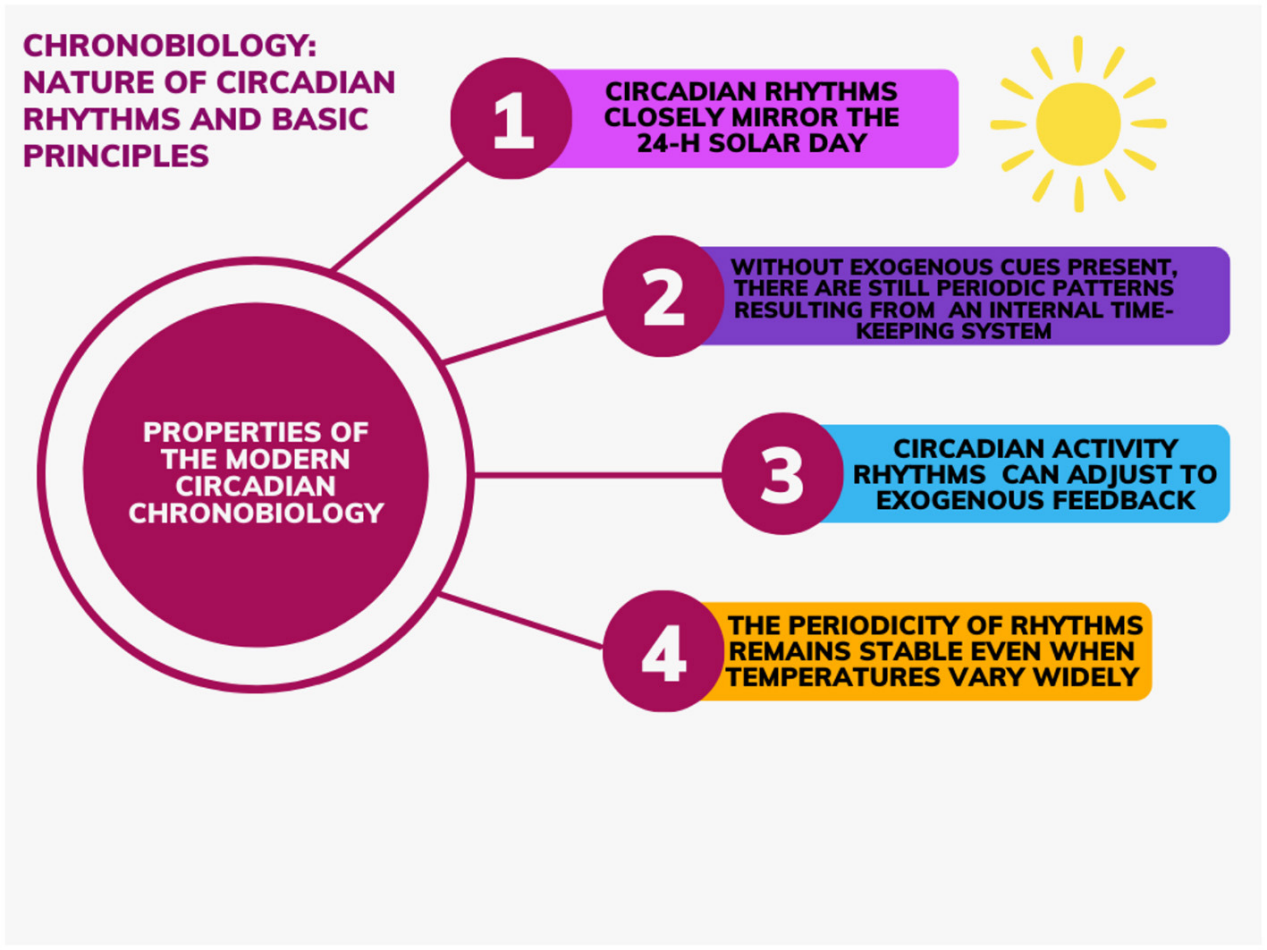
Properties to the bases of modern circadian chronobiology.

Since 1729 and the discovery of the presence of circadian rhythms maintained even with deprivation of all temporal indices, studies with temporal isolation protocols have allowed experts to deduct and attribute distinct/unique properties to the bases of modern circadian chronobiology. The notions (see Figure 1) can be summarized as follows:

1) Circadian rhythms closely mirror the 24-h solar day. Said period can be marginally shorter or longer (±0.55% of variation in humans) (Czeisler et al., 1999). The low variability in circadian period in classical free-living conditions suggests that the intrinsic period of the human circadian pacemaker is under impressive tight genetic control, as widely demonstrated in other species (Czeisler et al., 1999);
2) Without exogenous cues present, there are still periodic patterns providing evidence that these rhythms are a result of an internal timekeeping system (self-sustained);
3) Circadian activity rhythms can adjust to exogenous feedback (e.g., light entrainment);
4) The periodicity of rhythms remains stable even when temperatures vary widely (temperature compensation) (Dunlap et al., 2007).

These principles of chronobiology highlight that circadian rhythms persist in environments free of external timing signals, following a near 24-h cycle length. A strong body of evidence demonstrates that a cell-autonomous program of gene expression accounts for the human circadian clock genes that produce these rhythms (Vitaterna et al., 2001). However, under normal living conditions, circadian rhythms are normally synchronized by environmental stimuli. The term “zeitgebers,” German for “time giver” is used to describe said external cues that entrain the human circadian rhythms (Grandin et al., 2006; Refinetti and Menaker, 1992). As an example, it was thought that the most prominent circadian rhythm is the sleep wake-cycle had a free-run period of approximately 25-h. However, those observations were derived from studies on humans exposed to light levels that interfered with accurately estimating circadian periods. New research involving precise measurements of melatonin, core body temperature, and cortisol rhythms in healthy young and older individuals under strictly controlled lighting conditions has shown that the intrinsic period of the human circadian pacemaker averages 24.18 in both age groups, with a consistent distribution similar to that found in other species (Czeisler et al., 1999; Mistlberger and Rusak, 2005). Along those lines, light is the dominant zeitgeber in most species with the potential of inducing phase shifts that can vary in magnitude and direction based on the circadian phase of exposure (Mistlberger and Rusak, 2005). It is now well recognized that daily rhythms are generated by endogenously rhythmic internal clocks and that every living photo sensible organism is subjected to cyclical changes in environment and have therefore developed behavioral, physiological, and metabolic rhythms oscillating on an approximate 24-h cycle, named the circadian rhythms (Zee, 2016). The following section will explore specific characteristics and parameters of circadian rhythms.

### Parameters of circadian rhythms

Virtually all physiological and biochemical processes follow a circadian rhythm (Richter et al., 2004). Circadian rhythms are found in intracellular functioning (i.e., gene expression and cell communication and reproduction) but also in tissue systems, organs, and their functions (Cauter et al., 1981; Koopman et al., 1989; Schibler, 2005; Zanello et al., 2000). A circadian rhythm is defined by various parameters: period [i.e., time required for the completion of a cycle (oscillation), approximately 24-h], amplitude, a measure of intensity that signifies the height of a peak in a cycle (i.e., the difference between the peak and the equilibrium or midpoint value, or half the peak-minus-trough value) (Palmer, 2012), and phase [i.e., position relative to a reference point in the cycle (e.g., beginning of the night phase)] (Refinetti and Menaker, 1992). In some cases, it is possible to denote a phase delay or a phase advance relative to the solar cycle or another astronomical time point. This can be explained by the fact that, although circadian rhythms are known to persist in the absence of external signals (free running), normally environmental cues are present and thus rhythms are aligned to said cues. Entrainment of the circadian rhythms in some cases (e.g., traveling across time zones) can either shift the phase back or ahead (Bruce, 1960; Schulz and Steimer, 2009). In humans, most of these processes are influenced by a complex circadian timing system in which the master biological clock regulates timing of most, if not all, 24-h behavioral, physiological, and biochemical rhythms (Rosenwasser and Turek, 2015). Said master circadian clock is in the bilaterally paired suprachiasmatic nucleus (SCN) of the anterior hypothalamus (Rosenwasser and Turek, 2015). The following section will discuss said master circadian clock.

### The suprachiasmatic nucleus: master biological clock

The central oscillator lies in SCN of the hypothalamus and is often interchangeably referred to as the central circadian clock, master biological clock or central/circadian oscillator/pacemaker. In humans, the SCN has been firmly recognized as the master circadian pacemaker atop a hierarchically organized and anatomically distributed circadian system which entrains downstream peripheral oscillators/clocks via neural and neuroendocrine pathways (Rosenwasser and Turek, 2015). There is substantial evidence to justify the SCN's reputable title as it is responsible for direct and indirect regulation of most, if not all, circadian rhythms in mammals (Vansteensel et al., 2008). Through *in vivo* and *in vitro* approaches, it has been made clear that the SCN is a complex heterogeneous neuronal network. Numerous SCN lesion studies have shown that, in mammals, the SCN can sustain rhythmic activity while maintaining clear rhythmicity in both central and peripheral tissues (Hastings et al., 2003; Silver and Schwartz, 2005). Indeed, ablation or surgical isolation of the SCN within the brain abolishes overt behavioral rhythmicity (Moore and Eichler, 1972). In the circumstance of experimental SCN transplant, rhythmicity was restored unambiguously in approximately 80% of the arrhythmic hosts receiving the SCN implants (LeSauter et al., 1996). The period of restored rhythms consistently matched the donor tissue's genotype. In addition to the ability of the SCN to ensure rhythmicity while the organism is free-ran, the SCN allows the organism to anticipate and oscillate according to the physical environment (i.e., synchronization to the environment). The SCN also provides internal temporal organization and ensures that intracellular changes occur in coordination with one another and in homeostasis with the environment (Vitaterna et al., 2001). At the cellular level, every cell generates an intercellular molecular cascade leading to a negative feedback loop with a period of approximately 24-h (Reppert and Weaver, 2001). This negative feedback loop is called “circadian molecular clock” and is managed independently by the cell. The cellular-molecular basis of circadian rhythm involves several circadian clock genes that are expressed in the SCN and throughout the brain, peripheral tissues, and organs (Rosenwasser and Turek, 2015). The next section will cover the genetic mechanisms associated with the biological clock and rhythms in humans.

### Genetic mechanisms underlying and associated with the biological clock and rhythms

**Figure 2:**
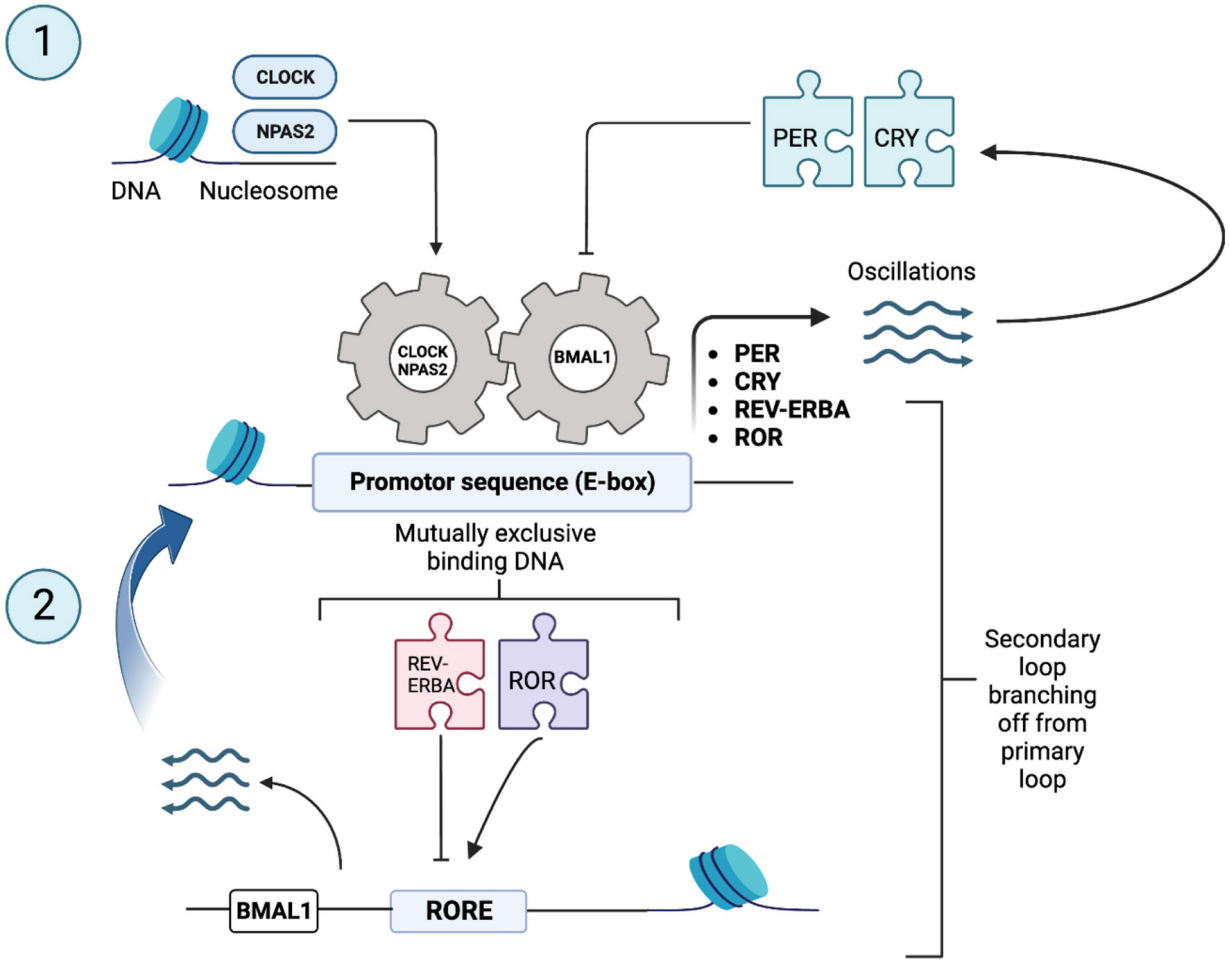
Core clock genes and canonical transcriptional feedback loops in core clock genes [Adapted from Reinke and Asher (2019)]. The body's molecular clock, found in nucleated cells, relies on a complex loop involving specific genes - often referred to as “clock genes.” These genes, including *Period* (*PER*) and *Cryptochrome* (*CRY*), are set in motion by proteins known as transcription activators. PER and CRY proteins are depicted as puzzle pieces in the figure to symbolize their role in regulation gene expression. The transcription activators, such as Circadian Locomotor Output Cycles Kaput (CLOCK), Neuronal Per-ARNT-Sim Domain Protein 2 (NPAS2), and Aryl Hydrocarbon Receptor Nuclear Translocator-Like Protein 1 (ARNTL1, commonly known as BMAL1), form combinations like CLOCK–BMAL1 and NPAS2–BMAL1. CLOCK, NPAS2, and BMAL1 are depicted as gears in the figure to symbolize their role in regulating the circadian clock. These pairs then latch onto E-box elements (an E-box is a DNA sequence found in some eukaryotic cells that serves as a site for protein binding. It plays a role in regulating gene expression in neurons, muscles, and various other tissues) in specific promoter regions, sparking the transcription of *PER* and *CRY* genes. PER and CRY proteins are subsequently ushered into the cell nucleus, where they act as repressors for their own genes' transcription. This action sets the stage for a new daily cycle to begin. Adding an extra layer of resilience to this oscillating mechanism is a secondary feedback loop. This loop involves nuclear receptors belonging to the Nuclear Receptor Subfamily 1 Group D member 1 (NR1D1, commonly known as REV-ERBA) and RAR-Related Orphan Receptor (ROR) families. These nuclear receptors, too, are spurred into action by components of the core clock oscillator, driving the rhythmic expression of *BMAL1* from sites known as ROR response element binding sites (RORE binding sites). These intricate feedback loops collectively orchestrate rhythms that occur with a roughly 24-h period and are thus termed “circadian,” derived from the Latin “circa diem” meaning “about a day.”

The SCN clock is composed of single-cell circadian oscillators that, when synchronized, regulate overt rhythms through coordinated circadian outputs. It is possible that all 20,000 cells that make up the SCN in mammals are, in fact, clock cells (Reppert and Weaver, 2001). Almost half of all protein-coding genes show circadian-dependent transcription in at least one tissue in humans and rhythmic expression of genes is accomplished through a cell intrinsic transcriptional/translational feedback loop (TTFL) (Zee, 2016). At the cellular level, circadian oscillations are driven via rhythmic expression of core clock genes and their protein products. The human molecular clockwork is composed of a core set of said regulatory clock genes involved in the TTFL including: (a) Period 1, 2, and 3 (*PER1, PER2*, and *PER3*); (b) Cryptochrome 1 and 2 (*CRY1* and *CRY2*); (c) Nuclear receptor subfamily 1 [*Nr1d1* (*REV-ERB-*α) and *Nr1f2* (*ROR-*β or *ROR-*γ)]; (d) Circadian Locomotor Output Cycles Kaput (*CLOCK* and *NPAS2*); and (e) Aryl hydrocarbon receptor nuclear translocator-like 1 and 2 (*BMAL1*) (Vitaterna et al., 2001; Hickie et al., 2013; Lowrey and Takahashi, 2011). *PER* and *CRY* genes are activated by the transcription activators: CLOCK, neuronal Per-Arnt-Sim domain protein 2 (NPAS2) and BMAL, which form two heterodimers CLOCK-BMAL1 and NPAS2-BMAL1 (Reinke and Asher, 2019). These complexes then bind to DNA promoter sequences, E-box elements, in promoter regions and allow for the transcription activation of *PER* and *CRY* genes (whose expression is rhythmic and typically peaks during the subjective night in the SCN of mammals) (Hickie et al., 2013; Koike et al., 2012). Once formed, PER and CRY proteins are imported in the cell's nucleus and act as negative regulators by directly inhibiting the transcription of their own gene loci (Reppert and Weaver, 2001; Hickie et al., 2013; Reinke and Asher, 2019). Therefore, this first negative TTFL allows for a new circadian cycle to begin (Figure 2). A secondary feedback loop contributes to the robustness of the oscillatory mechanisms/biological rhythms. This loop consists of nuclear receptor subfamily 1 group D member 1; commonly known as Nr1d1 and RAR-related orphan receptor (ROR) families, which, once activated by components of the core clock oscillators, also drive rhythmic BMAL1 expression from ROR response element binding sites (RORE). The ROR transcription factors stimulate BMAL1 transcription, and REV-ERA transcription factors suppress it. This second feedback loop modulates the transcription of the first feedback loop. These complex loops generate an autonomous rhythmicity with a period of about a day (i.e., 24-h circadian rhythms) (King and Takahashi, 2000). Thus, gene expression is central to the generation of circadian rhythms. Almost every individual cell (e.g., fibroblasts and muscle and fat cells) and therefore organ system (e.g., liver, pancreas, and gut) have their own intrinsic clocks and oscillatory mechanisms at the cellular level- referred to as peripheral clocks (Yamazaki et al., 2000). Said cellular and organ-based clocks from different physiological systems need to be aligned in coherent patterns and the master circadian clock synchronizes and drives this alignment to key behavioral, and physiological rhythms (Hickie et al., 2013; Mohawk et al., 2012). Assessment of peripheral circadian rhythms are measured via gene expression of peripheral cells (Nováková and Sumová, 2014). As such, circadian gene expression is widespread throughout the body yet input from the SCN (also composed of clock cells functioning by negative feedback loop) *in vivo* is still essential. This demonstrates that rhythmic gene expression can be driven both intrinsically and by extracellular cues through SCN control: ultimately promoting optimal functioning (Mohawk et al., 2012).

### Gene input and output regulation

**Figure 3:**
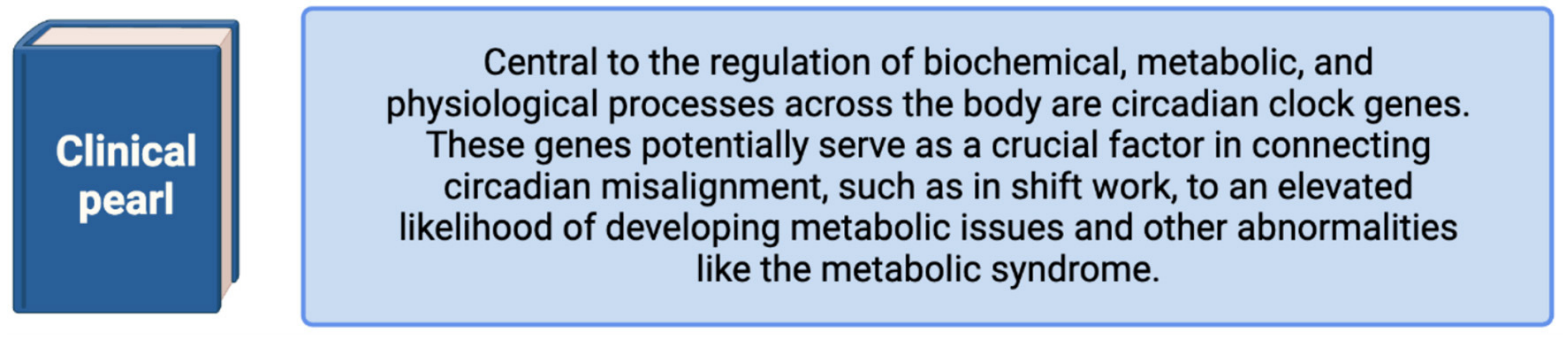
Clinical pearl.

Input gene products [e.g., Melanopsin (member of the opsin family of photopigments was first found in the inner retina) and *RAB3A* gene (identified in a mutagenesis screen)] detect external stimuli and then relay the input to the central oscillator to either reset, synchronize, or entrain it. They include photoreceptors designed specifically for regulating the clock and are expressed as proteins varying in origin, characteristic, and function (Vitaterna et al., 2001; Cermakian and Sassone-Corsi, 2000). Output gene products (clock-controlled genes), which encompass peptides and transcription factors (i.e., proteins that control gene expression) convey rhythmic information in hierarchal manner downstream to physiological systems (Cermakian and Sassone-Corsi, 2000; Vitaterna and Turek, 2010). The central circadian oscillator is synchronized by a highly complex molecular machinery including clock factors that drive rhythmic expression of target genes outside the core clock-controlled genes, whose exhaustive description is beyond the scope of this paper [please refer to “Crosstalk between metabolic and circadian clocks” by Reinke and Asher (2019), and/or “Principles and Practice of Sleep Medicine” – 5^th^ edition by Kryger et al. (2010)]. Overall, the molecular clockwork involves TTFL containing both positive and negative elements (Shearman et al., 2000). These molecular loops lead to the display of outstandingly precise 24-h rhythms in metabolic (e.g., glucose and lipid metabolism) (Duez and Staels, 2009), physiologic (e.g., hormones such as melatonin and corticosteroids) (Gooley and Saper, 2005; Rosenwasser and Turek, 2017), and behavioral (e.g., feeding, activity levels, and sleep-wake pattern) rhythms in humans (Rosenwasser and Turek, 2015). A concrete example of this is related to individual chronotype, defined as an individual's preference of sleep/wake timing (Takahashi et al., 2008), which is thought to be genetically determined (Kalmbach et al., 2016). An association has been identified between an evening chronotype (delayed sleep circadian phase) and 3111C allele of *CLOCK* gene, while a morning chronotype (advanced sleep circadian phase) has been associated with polymorphism of the *PER1, PER2*, and *PER3* genes (Kalmbach et al., 2016; Kunorozva et al., 2017). However, a recent study demonstrated that genetic chronotype can be shifted by the impact of socio-environmental factors (Kunorozva et al., 2017). Together, this ultimately suggests that chronotype is influenced by genetic, environmental, and social factors which reinforces the statement that genetic, physiological, and behavioral mechanisms all play a non-negligible role in circadian rhythms (Figure 3). With this in mind, the following section looks at the physiological mechanisms involved with the biological clock and biological rhythms in humans.

### Physiological mechanisms underlying/associated with the biological clock and rhythms

The internal time keeping system has been established as a hierarchical, interconnected network of clocks. The core molecular loop can be entrained by environmental cues to generate a rhythm through various regulated output pathways (downstream oscillators in the brain and periphery) (Rosenwasser and Turek, 2017) which successively regulate key biological functions (Bae et al., 2019). The human circadian system and the synchronization/entrainment of the SCN and peripheral oscillators can be considered in three components: the input pathways to the self-sustained SCN, the internal SCN regulatory system, and the output pathways by which the SCN regulates overt rhythms in biochemistry, physiology, and behavior (Kalsbeek et al., 2006).

### SCN input (entrainment) regulation pathways: photic input pathways

Important contributing stimuli to phase control within the SCN include internal synchronizers (e.g., hormonal signals) and external synchronizers, such as the light, which has been evidenced as the main zeitgeber for circadian entrainment (via input pathways) and critical to setting the biological clock to the 24-h light/dark cycle in most mammals (Roenneberg et al., 2007). The SCN has three input connections, the most important consisting of the retinohypothalamic tract (RHT), allowing afferent projection of photic information (Morin, 2013). The other functional inputs to the circadian clock include the median raphe projection and the geniculohypothalamic tract from the thalamic intergeniculate leaflet (Rosenwasser and Turek, 2017). The latter two have been described as secondary, indirect pathways that are not crucial for photic entrainment of the SCN (Morin, 1994). It has been established that the RHT, a specialized retinal projection system, is crucial and sufficient for photic entrainment of the biological clock (Reppert and Weaver, 2001; Rosenwasser and Turek, 2017; Johnson et al., 1988). In humans, photoreception is said to be based entirely on retinal mechanisms within the eye because bilateral enucleated (enucleation refers to a surgical procedure where the entire eyeball is extracted) mammals are incapable of light entrainment (Rosenwasser and Turek, 2017; Foster et al., 1991). However, through retinally degenerate mammals (C57BL/6J mice and hamsters) studies, it has been demonstrated that in the absence of classic photoreceptors, such as rods and cones, ordinary circadian entrainment responses to light remain (Reppert and Weaver, 2001; Johnson et al., 1988; Foster et al., 1993). This led to the discovery of the intrinsically photosensitive non-cone photoreceptor system, such as the retinal ganglion cells using the peptide melanopsin as a photopigment (Rosenwasser and Turek, 2017; Hattar et al., 2003). Melanopsin expression was evidenced to be present in the human retina and involved in photoentrainment of both “normal” and blind individuals (Hannibal et al., 2004). Melanopsin-containing ganglion cells, members of the opsin family of proteins, contribute, but are not necessary, to light entrainment via the RHT to the cells of the SCN. Indeed, SCN photic entrainment is only completely abolished when both classical rod-cone and melanopsin-based photoreception have been eradicated (Hattar et al., 2003). Together, they are essential for relaying the external light inputs to the biological clock and consequently entraining biological rhythms in humans (Vitaterna and Turek, 2010). For example, when light exposure occurs at the beginning of the subjective night, light pulses induce a phase-delay shift in circadian rhythms whereby the onset of the next subjective day is consequently reset to a later time where said circadian rhythms resumes it's free-run state from this new phase (Mistlberger and Rusak, 2005; Lee et al., 2003).

### Synchronization of the organism to the oscillations of the master biological clock

In addition to being the primary target of the RHT, the SCN is the target of several multiple other inputs from the cortex, the limbic system, and visceral organs and receives direct monosynaptic projections from at least 35 different brain regions; providing a wide range of extrinsic/intrinsic stimuli and, in turn, revealing enormous potential for circadian pacemaker modulation (Morin, 2013). Indeed, the SCN will integrate this information and adjust its own oscillations to deliver different waves of SCN transmitter release to several specific SCN target areas (Kalsbeek and Buijs, 2002). This will affect both neuroendocrine mechanisms and the peripheral autonomic nervous system (Kalsbeek and Buijs, 2002). Moreover, the specialization of the SCN appears to contain neurons specifically targeting the liver, pineal and adrenal gland (Kalsbeek et al., 2006).

### SCN output regulation pathways

The molecular mechanisms underlying the function of the biological clock consist of genetic feedback loops in which proteins downregulate their own transcription and stimulate the transcription of other clock proteins (Kalsbeek and Buijs, 2002). Once the molecular rhythm is made, connections between the SCN neurons and specific SCN targets are essential for the whole transmission of the circadian rhythms from the biological clock (Kalsbeek and Buijs, 2002). Based on morphology and neurotransmitter phenotype, the SCN can be divided into two components: the dorsomedial (SCDNdm) and ventrolateral (SCNvl), referred to as shell and core, respectively (Gooley and Saper, 2005). The shell SCN output reaches the dorsomedial nucleus of the hypothalamus (DMH) and has three different targets: (1) the ventrolateral preoptic nucleus (VLPO) which regulates sleep; (2) the lateral hypothalamus which regulates wakefulness and feeding behaviors; and (3) the paraventricular nucleus (PVN), which regulates circadian hormonal secretion of melatonin and corticosteroids (Takahashi et al., 2008; Lee et al., 2003; Kalsbeek and Buijs, 2002; Saper et al., 2005). The core region of the SCN receives light input through the RHT. Neural and humoral signals serve as output signals from the SCN to other regions of the brain and the periphery (Takahashi et al., 2008; Saper et al., 2005). The SCN output pathways are directly and indirectly responsible for the timing of physiological functions [i.e., hormone secretion (melatonin and corticosteroids), sleep-wake cycle, feeding behavior, and thermoregulation] (Takahashi et al., 2008). The core SCN output reaches the sub-paraventricular zone (sPVz), which is then relayed to the medial preoptic region (MPO) to control circadian rhythms of core body temperature (i.e., thermoregulation) (Saper et al., 2005). The PVN also indirectly receives input from core SCN outputs via the sPVz to the DMH (Takahashi et al., 2008). Regarding the sleep-wake rhythm, cell-specific lesions to the sPVz resulted in elimination of sleep, locomotory activity, and core body temperature circadian rhythms signifying that SCN-generated circadian rhythms primarily rely on the sPVz's neuronal pathway (Lu et al., 2001). A second-order projection to the DMH also acts as a major neuronal pathway for SCN-generated circadian rhythms. In fact, the DMH plays a crucial role in integrating both photic and non-photic cues to establish a circadian sleep-wake cycle rhythm via its projections of inhibitory neurotransmitters thought to promote sleep to brain areas involved in regulating sleep and wakefulness (Gooley and Saper, 2005). Since the SCN is in the brain, assessment of its functioning is done via indirect measures of the circadian rhythms/substrates it produces (Nováková and Sumová, 2014). When measuring circadian markers, it is crucial to control for external cues/behaviors (e.g., physical activity or food consumption) and other physiological phenomena that could interfere (masking effect) with the expression of these internal circadian rhythms for ensuring that the endogenous rhythms are properly being measured. Almost all rhythmic physiological functions could be used as indirect measures of the SCN (Nováková and Sumová, 2014). Behavioral circadian rhythms related to scheduled feeding, activity/arousal states, and social cues are also said to be valid and important markers of human circadian rhythms (Monk, 2010). However, the crucial need to control masking is a size limiting factor, as is the need to take several measures during a physiological unit of time lasting 24-h (Schulz and Steimer, 2009). Thus, core body temperature, melatonin, and cortisol levels are often used as classic/optimal markers of human circadian rhythms (Nováková and Sumová, 2014; Monk, 2010).

Neuroendocrine rhythms are linked to the connection between the rhythmic activity generated in the SCN and the rhythmic release of melatonin from the pineal gland (Kalsbeek et al., 2006). Melatonin (detected and measured in saliva and blood samples) is often the gold standard marker of the circadian system (Nováková and Sumová, 2014). Its secretion from the pineal gland is directly controlled by the SCN and provides the organism with information about the time of day, as it follows a robust secretion rhythm at night. Light input via the RHT activates SCN neurons and lead to a dose-dependent inhibition/suppression of melatonin secretion (Nováková and Sumová, 2014; Gooley and Saper, 2005). Conversely, melatonin is produced during the subjective night (i.e., when SCN activity is low) (Gooley and Saper, 2005). Once secreted, melatonin reaches many different targets in the central nervous system, including the SCN as well as peripheral targets. Specifically, melatonin coordinates peripheral oscillators to the light-dark cycle while also inducing sleep onset, promoting optimal sleep architecture, and synchronizing other physiological and behavioral aspects of the sleep period (Hickie et al., 2013). In turn, melatonin feeds back directly to the SCN via melatonin receptors (MT1 and MT2) in the SCN, inhibiting SCN electrical activity for increasing wakefulness (Hickie et al., 2013; Gooley and Saper, 2005). Thus, endogenous melatonin is an essential hormone in the regulation of human central and peripheral circadian rhythms. Accordingly, the use of exogenous melatonin has become increasingly popular as a means of promoting resynchronization in situations of circadian disruption (Burke et al., 2013). In the following section, the main behavioral mechanisms that contribute to the synchronization/maintenance of biological rhythms in humans will be discussed.

### Behavioral mechanisms underlying and associated with the biological clock and rhythms

Circadian rhythms in humans can be synchronized/entrained to the environment through non-photic zeitgebers (Mistlberger and Rusak, 2005). A true zeitgeber can be identified as a stimulus that affects the pacemakers rather than only overt behavior (Mistlberger and Rusak, 2005). Among the many behavioral non-photic stimuli, very few have been rigorously corroborated as functioning zeitgebers (Mistlberger and Rusak, 2021). Three of the main behavioral/non-photic zeitgebers will now be discussed.

### Activity levels and arousal states

The effects of behavior on the operational properties of the circadian pacemaker were not recognized until the late 1980s, when locomotor activity and arousal reliably induced large phase shifts in mammals (Mistlberger and Rusak, 2005). As with photic stimuli, timing of physical activity dictates the magnitude and direction of the phase shifts, and the effect of locomotor activity is dose dependent (Hughes, 2018). In humans, early evening exercise of high intensity (working intermittently [4–30 min] at 80%−95% of maximal rate of voluntary oxygen consumption) increases arousal and induce phases advances of circadian rhythms. Conversely, exercise performed at midnight induces phase delays in melatonin onset for example (Lewis et al., 2018; Buxton et al., 2003). Indeed, physical activity can lead to phase advances or delays in rhythms such as core body temperature, melatonin and thyroid stimulating hormone, and in the long term, optimal performance timing (Lewis et al., 2018). Unlike other non-photic behavioral zeitgebers, scheduled physical activity under a light-dark cycle can synchronize the phase of overt circadian rhythms by altering endogenous rhythms generated both by the SCN and peripheral tissues (i.e., adrenal glands, skeletal muscle, and liver) (Hughes, 2018). As an example, physical activity acts directly on the SCN by acutely suppressing its electrical activity and consequently increasing the amplitude of SCN rhythms and clock gene expression (Hughes, 2018). It has been proposed that exercise, at the right time, could promote chronobiological homeostasis and act as a health and performance enhancer (Lewis et al., 2018). The answer to the question of “how” exercise acts as a zeitgeber is unclear, but it is hypothesized that exercise modifies the internal environment and allows systemic signaling to circadian coordinating centers in the brain (e.g., myokine circadian rhythm) (Lewis et al., 2018; Perrin et al., 2015).

### Food and feeding behaviors

**Figure 4:**
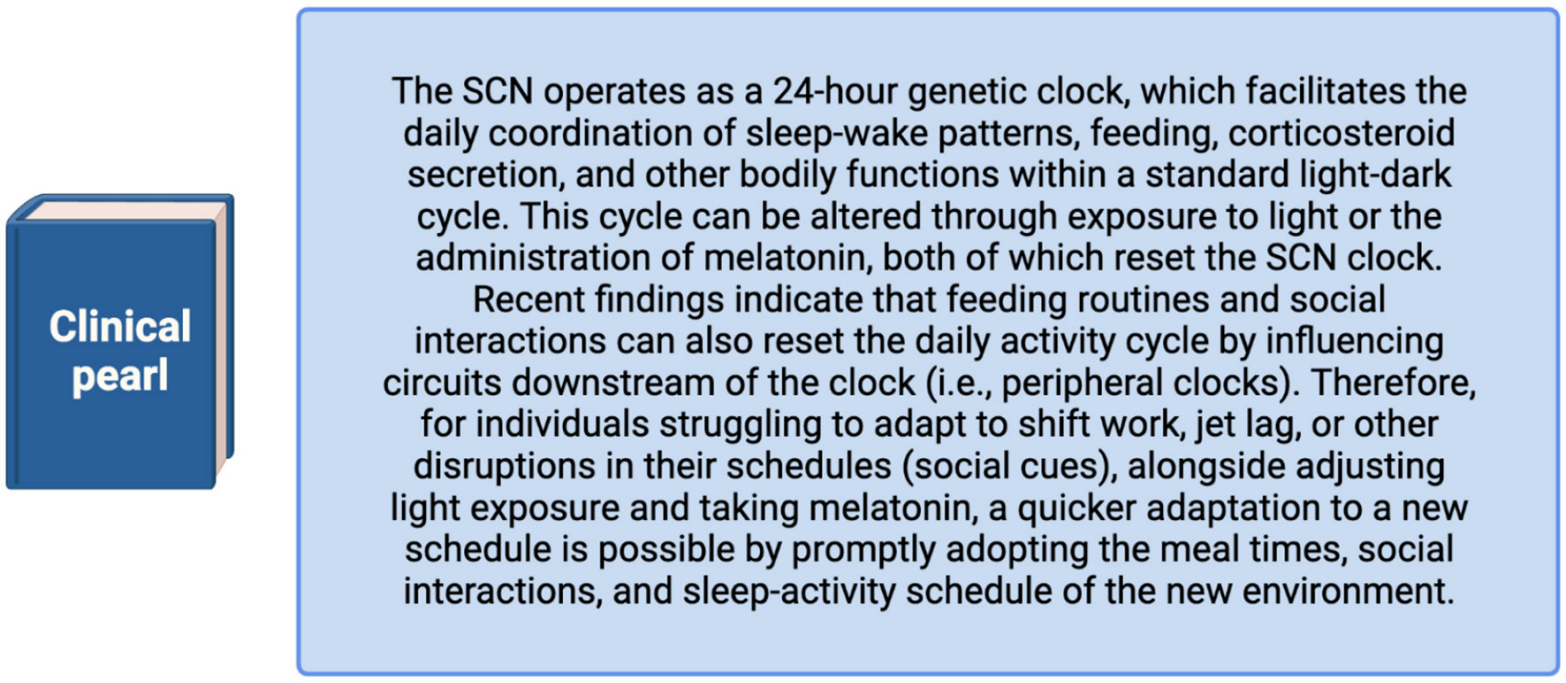
Clinical pearl.

The effect of food availability on the timing of circadian rhythms is demonstrated by studies in which access to food is restricted to a particular time of day. In a laboratory setting, mammals exposed to restricted feeding schedules led to a daily rhythm of food-anticipatory activity which seems to be controlled by peripheral rather than central (SCN) circadian clock (Mistlberger and Rusak, 2021). Anticipatory activity in preparation for mealtime includes increased wakefulness, locomotor activity, body temperature, and corticosteroid secretion (Mistlberger and Rusak, 2021; Gooley et al., 2006). SCN lesions did not lead to cessation of food entrainment, indicating that the master biological clock is not necessary for food entrained rhythms (Gooley et al., 2006). Conversely, the majority of peripheral organs (e.g., liver, kidney, and pancreas) express genes with a circadian rhythm that are preferentially entrained by the feeding cycle rather than light-dark cycles (Schibler et al., 2003). If food consumption occurs outside of the usual active phase, the peripheral oscillators will synchronize to feeding while the SCN will remain coupled to the light-dark cycle, causing a rhythm misalignment between SCN and peripheral oscillators (Reinke and Asher, 2019; Bae et al., 2019). With that said, through metabolic signals modulated by food consumption or restriction, peripheral tissues communicate to the SCN and modify output of the clock (Reinke and Asher, 2019). An example of this is related to a liver- derived starvation hormone (fibroblast growth factor 21) that binds to receptors in the SCN and alters circadian behavior to modify glucocorticoid secretion and alter circadian locomotor activity; features of adaptive starvation response (Bookout et al., 2013). Moreover, the SCN is bidirectionally connected to other hypothalamic nuclei, which relays metabolic states to the SCN and therefore regulates feeding behavior and synchronizes peripheral organs following food consumption (Reinke and Asher, 2019). In mice and hamster studies, it has been demonstrated that time-restricted feeding to the activity phase prevents obesity, hyperinsulinemia, and hepatic steatosis (Hatori et al., 2012; Salgado-Delgado et al., 2010). Although these studies were examining rodents, it provides insight for strong hypotheses around human rhythmic behaviors (Mistlberger and Rusak, 2005; Bae et al., 2019). This is a pertinent topic for consideration not only by those engaged in chronobiological research, but also by those working in the medical field. It offers a distinctive non-pharmacological approach, namely chrononutrition, which has the potential to inform the prevention and management of obesity, diabetes and other metabolic disorders that have been overlooked so far (Figure 4) (Reinke and Asher, 2019).

### Social cues

Social cues (e.g., shift work, jet lag, daylight savings, and school/work start times) can entrain the human biological clock and cause phase shifts in free-running rhythms (Mistlberger and Rusak, 2005; Roenneberg et al., 2007; Lack and Wright, 2007). However, social effects on circadian timing appear to be weak. This is evidenced by blind humans who follow regular work schedules while their biological clock is free-ran with its own 24-h periodicity (Roenneberg et al., 2007). Indeed, social cues alone cannot entrain the human circadian clock without concomitant light input, which complicates attributions of causality (Mistlberger and Rusak, 2021) and leads to serious problems in interpretating these effects (Mistlberger and Rusak, 2005). However, behavioral and social lifestyle can have a significant negative impact on human health via circadian misalignment with the environment. Lifestyles associated with abnormal exposure to zeitgebers disrupt the sleep-wake rhythm due to natural delays and impediments in the resynchronization of circadian clocks to shifted behavioral and environmental cycles (Mistlberger and Rusak, 2005). Shiftwork that involves circadian disruption has been classified as a carcinogen while also contributing to psychological, metabolic, cardiovascular, and sleep diseases (Lewis et al., 2018; Bae et al., 2019). Also, when sporting competitions require trans meridian travel, jet lag can lead to circadian misalignment to environment markers leading to competition times which do not match an athlete's optimal performance time (Reilly et al., 1997). Accordingly, there is a growing interest among health, scholar, and sport professionals to elucidate the factors that modulate the response to these social cues and develop potential strategies (i.e., light therapy or exogenous melatonin supplementation) to promote resynchronization following, for example, jet lag and shift work (Figure 4) (Burke et al., 2013). It is proposed that social cues can reset circadian rhythms by acting on circuits that are downstream of the SCN, but the mechanisms are not known/fully understood, and much work remains to gain a clear understanding (Mistlberger and Rusak, 2005; Gooley and Saper, 2005).

Finally, interactions between photic and non-photic zeitgebers (e.g., light and exercise) are both relevant and complex; depending on their relative timing and magnitude they may act synergically or mutually inhibitory in producing phase shifts in circadian rhythms. The integration of multiple zeitgebers by the master biological clock and peripheral oscillators allow for stable entrainment to the environment (Mistlberger and Rusak, 2005).

## Conclusion

There are significant genetic, physiological, and behavioral mechanisms involved in the regulation/synchronization of the biological clocks and rhythms in humans. Much progress has been made in the field of chronobiology, but, research gaps remain, such as the role of certain genes in the circadian system that have yet to be determined (Vitaterna and Turek, 2010), the extent of the impact of circadian desynchrony on health and disease, including in the context of safety, performance, and productivity (Rosenwasser and Turek, 2017), and the potential of other non-pharmaceutical stimuli (environmental or behavioral) on the synchronization of the biological clock and rhythms (Adamovich et al., 2017). These are questions that remain to be answered, always considering the intricate web of genetic, physiological, and behavioral mechanisms involved in the biological clock and rhythms in humans.

### Perspective

**Figure 5:**
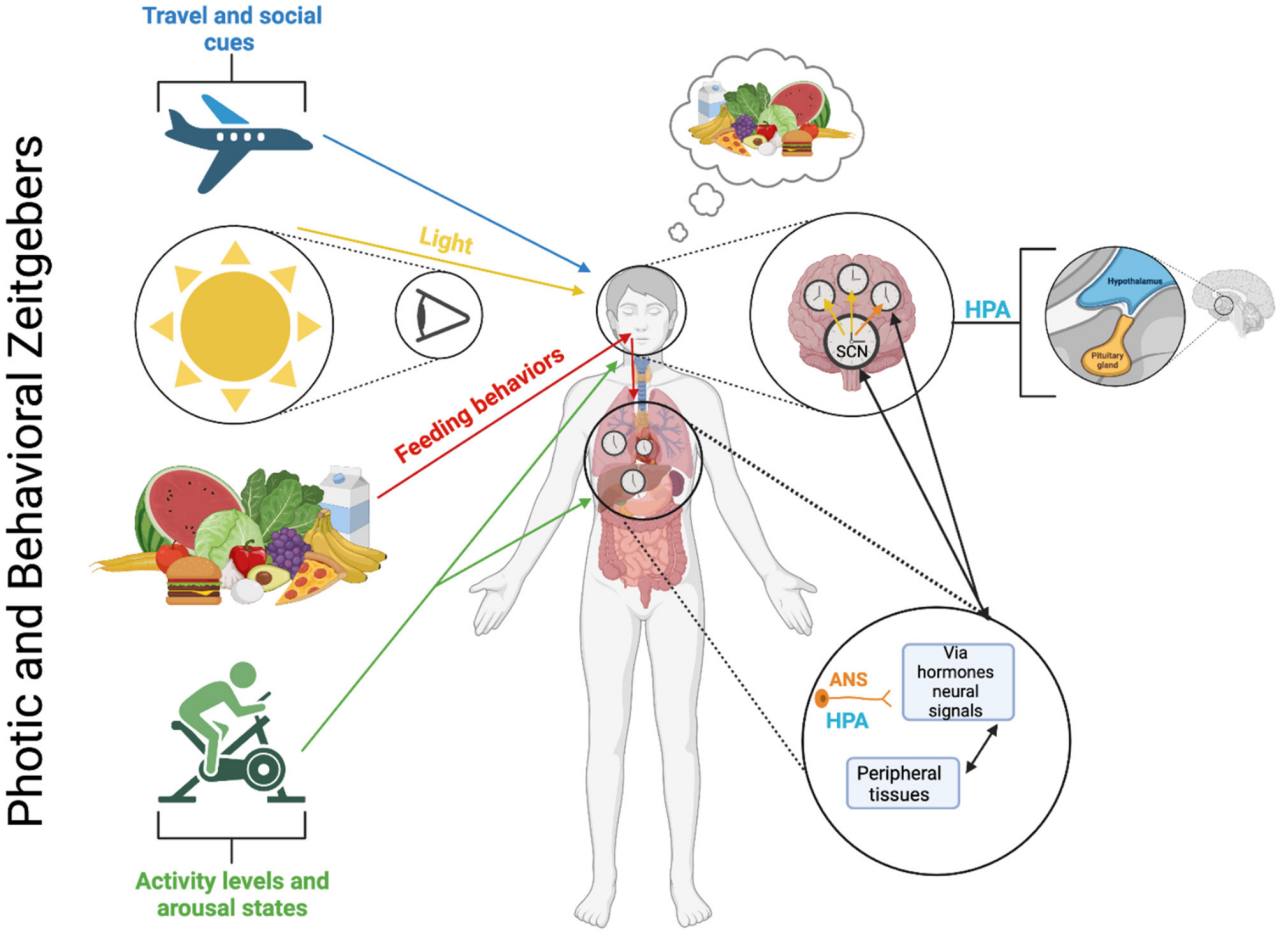
Photic and behavioral zeitgebers and the multioscillatory circadian timing system. The circadian timing system, which consists of several autonomously rhythmic circadian clock cells distributed in both central and peripheral tissues, operates through molecular feedback loops that control gene expression and cellular processes within each clock cell (refer to Figure 2). These molecular loops rely on similar, but not necessarily identical genes and proteins in different tissues. The central circadian pacemaker is in the SCN and is synchronized by light-dark cycles and environmental cues/behavioral mechanisms (i.e., activity levels, feeding behaviors, and social cues [note: specific mechanisms behind the social cues zeitgeber are not currently known or fully understood]). Within SCN neurons, cellular oscillators exhibit strong intercellular coupling, ensuring phase synchrony between cells and robust self-sustaining rhythmicity at the tissue level. However, under certain conditions, such as constant light, these coupling relationships can weaken, leading to a loss of coherent rhythmicity at the tissue (and behavioral) levels. Cellular oscillators also contribute to tissue-level rhythmicity in genomic and physiological processes in neural tissues and various peripheral tissues and organs. Generally, cellular oscillators located outside the SCN exhibit weaker coupling than those within the SCN. Consequently, they rely on rhythmic inputs from the central SCN to uphold phase synchrony. Without input from the central SCN pacemaker, a lack of phase synchrony at the cellular level can lead to a reduction in rhythmicity at the tissue level. The combined activity of the SCN and non-SCN central neural oscillators influences rhythmic behaviors like food intake and motor activity, autonomic nervous system (ANS) function, and hormone secretion from the hypothalamic-pituitary-adrenal (HPA) axis. These behavioral and physiological rhythms, in turn, generate additional rhythmic signals such as glucose availability, corticosterone levels, and body temperature, maintaining phase synchrony among peripheral oscillators in a likely tissue-specific manner. Furthermore, the activity of peripheral oscillators can produce rhythmic signals (e.g., peripheral hormones, autonomic afferents, and metabolic signals) that contribute to the synchronization of the SCN pacemaker and other central oscillators.

Societal and economic pressures, such as shift work, and technological advances, such as artificial lighting and increased screen time, expose us to conflicting environmental signals that disrupt our internal clocks. This desynchronization occurs when peripheral rhythms in various tissues and organs are no longer in sync with the central circadian clock. Key external synchronizers like light exposure and the sleep-wake cycle can easily become misaligned, leading to circadian desynchronization, particularly in cases of jet lag or shift work (Figure 5) (Kuhlman et al., 2018; Gooley et al., 2006). Shift work specifically, for example, affects over 30% of Canadian workers, including many healthcare professionals, and is known to disrupt not only the sleep-wake cycle but also other physiological rhythms that maintain bodily equilibrium (Shields, 2002). The resulting misalignment is linked to heightened fatigue and a range of physical and mental health risks (Salgado-Delgado et al., 2010). Given the growing impact of circadian misalignment on health, there is an increasing need for effective strategies that can help restore synchronization, particularly for those affected by shift work, jet lag, and other circadian-related disorders. While current chronotherapeutic methods face several challenges (as discussed below), recent advances offer promising solutions to address these issues.

Effective chronotherapeutic strategies to promote resynchronization are still limited, often facing challenges related to factors such as dosage, timing, accessibility, and inconsistent efficacy (Cheng et al., 2021; Bin et al., 2019). However, a validated intervention that could overcome these barriers would have significant clinical and practical applications for conditions such as jet lag, shift work disorders, and circadian-related sleep disorders. Recent advances in chronotherapy, such as targeted light exposure (luminotherapy) combined with exogenous melatonin, have shown promise in helping to realign circadian rhythms and improve overall health (Cheng et al., 2021). With the growing interest in chronointerventions, such as chrononutrition (i.e., meal timing for optimal health and entrainment of our body clocks) (Oda, 2015) and chronomedicine (also referred to as chronotherapeutics; i.e., administering medication at specific circadian times to enhance efficacy) (Smolensky and D'alonzo, 1993), we are gaining valuable insight into how these modalities—such as luminotherapy combined with melatonin—can support circadian alignment and promote better health. This progress highlights the critical importance of timing in the efficacy of proposed interventions. One notable example is the discovery that the timing of certain medications, rather than just the dose, can improve therapeutic efficacy. Indeed, the circadian structure highlights its significant importance for medical practice and patient pharmacology (Smolensky and D'alonzo, 1993; Smolensky and Peppas, 2007; Tahara and Shibata, 2014). One of the key objectives of medical chronobiology is chronotherapeutics—improving the effectiveness of treatments by taking into account the interaction of medications with the body's natural rhythms and the predictable changes in disease patterns and severity over time (Smolensky and Peppas, 2007; Kaur and Bala, 2013). In summary: one of the main goals of medical chronobiology is to optimize pharmacotherapy through chronotherapeutics (Smolensky and D'alonzo, 1993). In line with this, several studies have emerged that provide evidence of increased drug efficacy following bedtime administration (Kaur et al., 2013; Ayala and Hermida, 2010; Bolk et al., 2010; Hermida and Ayala, 2009). To cite a few specific examples, there has been improved efficacy in blood pressure control and thyroid hormone levels with the administration of various drugs (e.g., aspirin, levothyroxine, torasemide, ramipril, olmesartan, etc.) when ingested at bedtime vs. morning or other randomized times (Kaur et al., 2013; Ayala and Hermida, 2010; Bolk et al., 2010; Hermida et al., 2008, 2009). Other studies have also emphasized the relationship between food and the circadian system. Chrononutrition is understood as involving two key elements: (i) the timing of food intake and how specific nutrients support overall health, and (ii) how the timing of eating or certain nutrients can quickly influence or reset our internal clocks. Similar to chronopharmacology, chrononutrition is expected to become an essential approach for maintaining health in sync with our circadian rhythms (Tahara and Shibata, 2014). An illustration of this was seen in research showing that morning chocolate intake (vs. evening chocolate intake), has different effects on metabolic and physiological parameters such as hunger and appetite, substrate oxidation, fasting glucose, microbiota, and sleep and temperature rhythms (Hernández-González et al., 2021). In a different context, physical activity has also been shown to affect both sleep and metabolic processes, with potential optimization of health benefits depending of the timing of exercise; however, little is known about the precise mechanisms and optimal timing for these effects (Gabriel and Zierath, 2019). Exercise is a potent modulator of skeletal muscle metabolism and plays a key role in the management of metabolic disease. Tailoring exercise to an individual's circadian rhythms appears to be a promising approach for combating metabolic disease and maximizing the health benefits associated with exercise (Gabriel and Zierath, 2019). For instance, as noted above, shift work has been associated with a number of adverse health outcomes including cardiovascular disease, diabetes, obesity, cancer, gastrointestinal ulcers, impaired cognitive function, and reduced general wellbeing. Shift workers are also at a higher risk for occupational incidents and often suffer from shift work disorder, characterized by insomnia, excessive sleepiness, and a shortened total sleep time (Sachdeva and Goldstein, 2020). Some research suggests that appropriately timed exercise has the potential to improve sleep various health markers of individuals at risk of metabolic diseases, such as shift workers (Easton et al., 2024). Indeed, some studies have recommended the introduction of physical activity during night shifts as a potential countermeasure to improve wellbeing and reduce health risks associated with shift work (Easton et al., 2024). Regarding the timing of exercise, some studies have found no significant changes in the assessed health parameters between morning and nighttime exercise (Saidi et al., 2021), while others have reported different autonomic nervous system responses depending on the timing of exercise (Yamanaka et al., 2015). Overall, it is becoming clear that large-scale randomized trials are needed to further explore the precise mechanisms behind how the timing of exercise, chrononutrition, and chronomedicine influence biological, physiological, and metabolic outcomes (Creasy et al., 2022). These studies could pave the way for harnessing the potential of chronointerventions through the strategic timing of exercise, nutrition, and medical treatments.

This emphasis on timing—the precise alignment of chronointerventions with an individual's circadian phase—signals a paradigm shift in health and medicine. A fundamental understanding of chronobiology, including genetic, physiological, and behavioral components, is essential for the development of more effective chronotherapeutic interventions. Once considered peripheral, sleep and circadian alignment are now recognized as primary pillars of health, alongside nutrition and physical activity. These concepts are rapidly becoming crucial elements in global health recommendations and are likely to transform how we approach health promotion and disease prevention. In conclusion, timing has emerged as a critical factor in chronomedicine and chrononutrition. This newfound understanding reinvigorates the fundamental principles of chronobiology, making them relevant and revolutionary when applied with precision. Ultradian rhythms, while less studied than circadian rhythms, play a significant role in various physiological and behavioral functions, such as the 90-min sleep cycle, growth hormone release, stress response, and eating patterns (Gerkema, 2002), and while interventions for ultradian and infradian cycles exist, further research is needed to fully integrate these rhythms into chronobiology and advance health interventions, ultimately enhancing the understanding and application of biological rhythms in improving patient care and public health. To take full advantage of these advancements, health professionals from all disciplines—whether nutritionists, dietitians, physicians, or public health specialists—need to be versed in the basic principles outlined in this review. Integrating these principles into the training and practice of health professionals is essential to improving patient care and public health, and the pursuit of pioneering chronobiological interventions is crucial for advancing both medical treatment models and individual health management strategies in the modern era.
